# Sensor-Enhanced Smart Gripper Development for Automated Meat Processing

**DOI:** 10.3390/s24144631

**Published:** 2024-07-17

**Authors:** Kristóf Takács, Bence Takács, Tivadar Garamvölgyi, Sándor Tarsoly, Márta Alexy, Kristóf Móga, Imre J. Rudas, Péter Galambos, Tamás Haidegger

**Affiliations:** 1 Antal Bejczy Center of Intelligent Robotics, Óbuda University, 1034 Budapest, Hungarysandor.tarsoly@irob.uni-obuda.hu (S.T.); peter.galambos@irob.uni-obuda.hu (P.G.); 2 John von Neumann Faculty of Informatics, Óbuda University, 1034 Budapest, Hungary; 3 University Research and Innovation Center, Óbuda University, 1034 Budapest, Hungary; 4 School of Computing, Queens University, Kingston, ON K7L 3N6, Canada

**Keywords:** robotic meat processing, smart gripper, gripping force control, slip detection

## Abstract

Grasping and object manipulation have been considered key domains of Cyber-Physical Systems (CPS) since the beginning of automation, as they are the most common interactions between systems, or a system and its environment. As the demand for automation is spreading to increasingly complex fields of industry, smart tools with sensors and internal decision-making become necessities. CPS, such as robots and smart autonomous machinery, have been introduced in the meat industry in recent decades; however, the natural diversity of animals, potential anatomical disorders and soft, slippery animal tissues require the use of a wide range of sensors, software and intelligent tools. This paper presents the development of a smart robotic gripper for deployment in the meat industry. A comprehensive review of the available robotic grippers employed in the sector is presented along with the relevant recent research projects. Based on the identified needs, a new mechatronic design and early development process of the smart gripper is described. The integrated force sensing method based on strain measurement and magnetic encoders is described, including the adjacent laboratory and on-site tests. Furthermore, a combined slip detection system is presented, which relies on an optical flow-based image processing algorithm using the video feed of a built-in endoscopic camera. Basic user tests and application assessments are presented.

## 1. Introduction

### 1.1. Background

**Figure 1:**
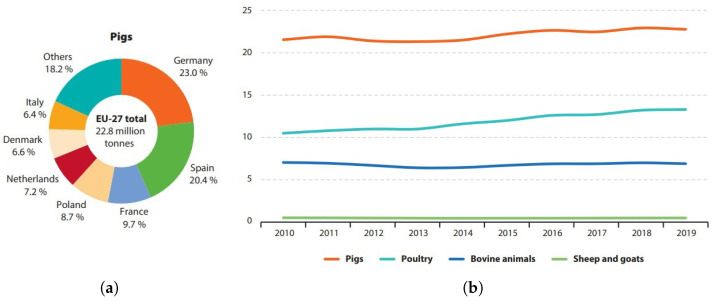
Livestock statistics from the European Union. (**a**) Distribution of pig meat production in 2019. (**b**) Production of different meat products in the EU, million tonnes. Source: Agriculture, forestry and fishery statistics [1]. Data source: Eurostat.

Food production and food processing are naturally some of the most important industries worldwide. With continuously increasing yields, the value of the produced output by the whole agriculture domain of the European Union (EU) in 2019 was around €418 billion, contributing 1.3% of its total GDP. However, the distribution of livestock is rather concentrated in the EU, e.g., 40% of the pigs are located in two countries, Spain and Germany (Figure 1a) [1]. This naturally results in centralized meat processing industries, which is beneficial from the point of view of automation and efficiency, but results in an unnecessarily large amount of transport for the animals and raises concerns regarding animal welfare.

Despite the increasing yields and economic indicators of the meat sector (Figure 1b), the volume of agricultural labor has been in stable decline in most states of the EU for decades. The average rate of labor input in the EU states between 2005 and 2019 was ~2.5% per year; however, this decline is even stronger in the Eastern European countries, e.g., ~13.5% in Bulgaria and ~8.4% in Hungary in 2019 [1]. These trends—the increasing yields with decreasing volume of labor—are most likely the results of the narrowing labor pool and act as a driver for automation as well, which shows that the latter is becoming a necessity in the agriculture sector. The spread of automation was further accelerated by the COVID-19 pandemic (especially in the meat sector) since hygiene became an even more important aspect and the sudden lack of labor force caused huge plants to close suddenly for weeks, even though some digital health technologies had been applied, such as hand hygiene [2,3,4,5,6,7].

Robots were originally used in factories with the goal of replacing human operators in repetitive, dangerous or physically demanding positions, and thus, object manipulation and gripping have been typical and important tasks of machines and robots since the beginning of industrial automation. By the 21st century, robots were introduced to most fields of industry; moreover, they appeared in other areas of modern life as well, such as surgical suits, restaurants, space research, etc. [8].

Despite all of these, the spread of robotics-based solutions and smart automated systems in the agri-food sector is remarkably slower than in most other industrial sectors. Although robots and machinery are regular at larger plants, decision-making typically remains the task of their human operators, i.e., intelligence is rarely embedded at the machine level. This is in part due to the lack of regulation and standards dedicated to this field (described in detail in [9]); meanwhile, the technical challenges are significant as well.

### 1.2. Meat Industry Grippers

In the food industry, particularly the meat sector, robotic grippers must handle soft, irregularly shaped, and slippery materials. Traditional grippers in the meat industry are robust, powered by pneumatic actuators or electromechanical motors, designed to manipulate heavy loads. However, the delicate nature of meat products requires intelligent grippers capable of precise control to avoid damage [10]. While soft grippers are gaining popularity, their current limitations in payload capacity and cleanability hinder their widespread adoption in meat processing.

Robotic grippers in the meat industry must be strong and powerful tools (especially in the red-meat industry, which is in the focus of this paper), since they need to manipulate huge masses of meat and body parts, such as whole legs of pigs or cattle. The required power—in the vast majority of cases—is provided by pneumatic actuators (typically cylinders) or electromechanical motors. The number of actuators, their size, placement and power level depend on the actual task as well; however, the cleanability and waterproofness of the EOAT are cardinal for every meat industry application. Although soft grippers (such as OnRobot’s SG-a-H, the Circular mGrip from Soft Robotics, SRT’s FG-FCA Circular Gripper or the grippers reviewed in [11,12]) are becoming increasingly popular for automated interactions between robots and soft objects, their (currently) relatively low payload and difficult disinfection prevent their direct utilization in the meat industry.

Other unconventional gripping principles also occur in the robot industry, and some of those can be utilized for meat industry automation too. Non-invasive gripping of large surfaces, for example, can be achieved through several different principles. Gecko-inspired adhesive gripping technologies are being researched, and the Gecko Single Pad Gripper from OnRobot is already on the market [13,14,15]. More conventional surface gripping technologies adopt chemical adhesives or electroadhesion; however, detachability and surface protection from damage or contamination are critical in this case [16,17]. Surface gripping can also be achieved by freezing the gripper and the target object if a sufficient amount of water is present; however, tissue damage due to freezing is an important issue. Penetrating gripping of soft target objects by hooks or spikes is simple and effective from a technical point of view; however, invasive techniques in the food industry are rarely allowed.

A widely used gripping approach in the meat industry is air-based gripping [10]. Several principles of fluid mechanics can be used to achieve robotic gripping (e.g., the Bernoulli principle [18], vortex-grippers [19], capillary effect [20] or the Coanda effect [21]), but only vacuum-grippers are strong enough for use in high-payload applications. Vacuum grippers usually consist of several suction cups, and their arrangement can be adjusted to the given task and target object. Furthermore, the holding force can easily be controlled through vacuum pressure, and since their cleaning is simple as well, vacuum grippers are widely used in the meat industry [10,22,23].

**Figure 2:**
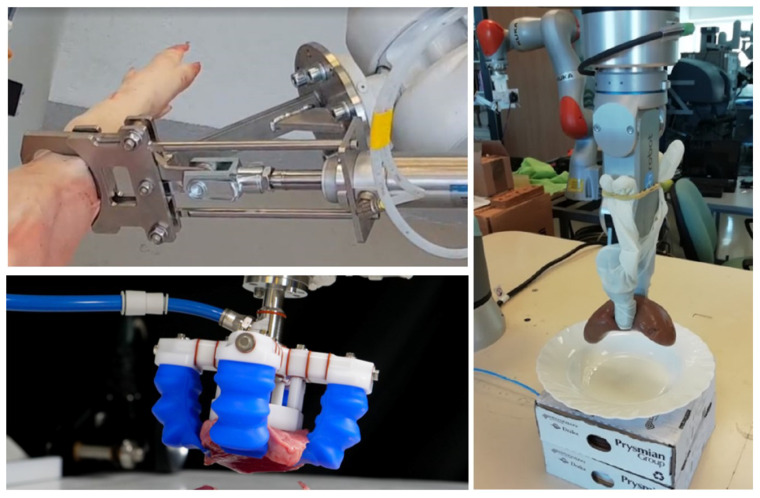
Examples of robotic grippers: simple strong pneumatic 2-finger meat industry gripper from DMRI (**top left**, image credit: DMRI), underactuated soft-gripper from Soft Robotics (**bottom left**, image credit: www.softroboticsinc.com, accessed on: 2 July 2024), and the force controlled OnRobot RG2 2-finger gripper delicately holding a pig kidney (**right**, image credit: Óbuda University).

In addition to vacuum grippers, the most common method in the meat industry is the fingers-based mechanical gripping (Figure 2 shows typical examples). These grippers usually consist of a base fixed to the robot flange and one or more mechanical finger(s) actuated by electromechanical motor(s) or pneumatic cylinder(s). The material and mechanical design of the finger(s) depend on the task of the gripper, but they have to be actuated at least along 1 DOF relative to the base of the gripper. The complexity of finger-based grippers extends from simple bistable mechanisms to intelligent five-finger robot hands; they may grasp with shape-locking or force-locking or may contain soft materials and underactuated mechanisms (Figure 2).

### 1.3. The RoBUTCHER Project

**Figure 3:**
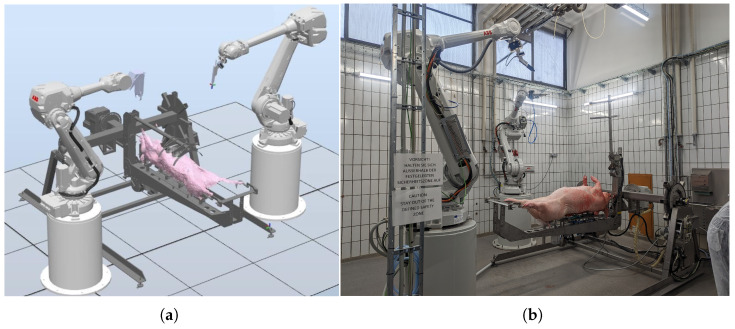
Early conceptual simulated setup of the Meat Factory Cell (**a**), and the real MFC at the Max Rubner Institute, Kulmbach, Germany (**b**). The Carcass Handling Unit is holding and manipulating the attached carcass, and the two robots are equipped with intelligent end-of-arm-toolings: RGB-D camera, knife/saw and gripper. (Simulation made with RobotStudio (v2021, ABB Ltd, Zürich, Switzerland), image credit: Óbuda University [28]).

To tackle the technical challenges of automated pig processing, the RoBUTCHER concept was born with a significant change in attitude toward the conventional line-based meat processing plants [24,25]. The Meat Factory Cell (MFC) designed within the RoBUTCHER project (see Figure 3) is a completely automated robot cell that should be capable of performing the main primary cuts of pig slaughtering, namely the cutting of the limbs and the evisceration process, i.e., the removal of the inner organs [26,27].

The robot cell consists of two industrial ABB robot arms, a motorized Carcass Handling Unit (CHU), intelligent cutting and gripping tools and an RGB-D camera fixed on one of the robots (Figure 3a). With the help of image-processing deep neural networks (trained with the data from professional butchers), the two robots autonomously move the intelligent cutting tools along the desired trajectories or the grippers to the desired gripping points. The separated parts of the carcasses (limbs, saddle, intestines, etc.) are placed on a rack inside the MFC, and thus, several instances of such cells may operate simultaneously (i.e., resulting in “parallel” meat processing plants instead of “linear” plants).

Reliably grasping and moving various parts of pig carcasses in such scenarios (i.e., automated meat processing) presents significant challenges. The anatomical diversity and variability in tissue consistency require grippers to exert substantial force for heavy parts like limbs, while also being delicate enough to handle slippery, fragile organs without damage. Residual soft tissue and fluids further complicate grip stability. Intelligent, sensorized grippers are essential to dynamically adjust gripping force and detect slippage, ensuring both meat integrity and process safety. These advanced technologies enable robots to perform complex tasks with high precision and reliability, addressing the stringent hygiene and efficiency demands of modern meat processing.

## 2. Materials and Methods

This section describes the design process and testing of a 2-finger intelligent gripper developed for general gripping tasks, particularly in the meat industry. The gripper is capable of grasping firmly but delicately different parts (soft tissues) of pig carcasses, but its mechatronical system can be scaled up or down for other animals too (e.g., lamb and cattle). The gripper’s main features are the following:

- Encircling gripping motion for target displacement tolerance;
- Cleanability, disinfectability, i.e., waterproof design;
- High payload (up to 30 kg);
- Fail-safe operation on power cut;
- Smart functionalities:
  - Force-controlled gripping;
  - Slip detection;
  - Wireless communication (for development and analysis);
  - Real-time feedback about successful grasping;
  - Real-time feedback about target displacement.

**Figure 4:**
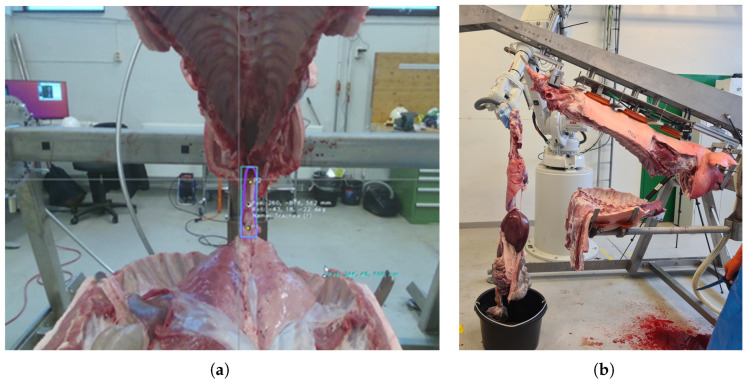
Images of a pig carcass fixed in the Carcass Handling Unit before and after the evisceration process. (**a**) Photo of the pig before the trachea-gripping taken by the RealSense RGB-D camera attached to one of the robots. The trachea and the gripping target point are successfully localized by the neural network (purple bounding-box and the labeled point in the middle). Image credit: ByteMotion. (**b**) The carcass in the CHU after the evisceration process. The limbs are already cut off, and the saddle, the belly and the viscera are separated and ready to be loaded onto the rack. Image credit: Óbuda University and NMBU.

The smart gripper was originally designed to handle the evisceration process in the MFC; however, the final version is capable of performing many more gripping tasks in automated meat processing plants. According to the MeaTable concept (described in detail in [26]), the evisceration process starts after the limbs are removed, the sides of the pig are cut along and the connective tissue below the spine is cut (see Figure 4a). In this state of the carcass, the trachea has to be grasped below the larynx, since all the inner organs (the pluck and the intestines) can be pulled out of the belly by the grasped trachea with only a few assisting cuts (see Figure 4b). The primary requirement is robust, image-guided grasping of soft objects, emphasizing that the robustness and reliability of the grasp are significantly more important than the quality of the grip.

In addition to the trachea gripping, the most usual gripping task during pig cutting is the manipulation of the limbs, which was the secondary focus during the gripper development process. In both cases, the gripping point and orientation in space are provided by image-processing AI software. A RealSense D435i RGB-D camera (Intel, Santa Clara, CA, USA) is attached to one of the robots and an imaging sequence provides a real-time digital twin of the pig carcass placed in the robot cell. The image processing software calculates the gripping point with an approaching vector (orientation) on the digital twin. Upon successful gripping target calculation, the central computer sends the target coordinates to the gripping robot and sends the closing command to the gripper after the robot reaches the target.

### 2.1. Requirements

In accordance with the description of the gripper’s task, the basic mechanical requirements and necessary functionalities were determined first. The gripper’s main purpose is to grasp the trachea and to reliably maintain a firm grasp throughout the whole evisceration process (Figure 4). This means that the robot has to be able to pull out the pluck and the intestines from the belly by the grip on the trachea, tearing the connective tissue between the viscera and the belly (only the diaphragm needs to be cut), which requires considerable load capacity.

If the cutting of the forelimbs and the side-cuts were carried out completely (Figure 4a shows such a case), then the target object to be grasped is only the trachea (together with the much smaller and softer esophagus), which is a cylindrical cartilaginous object with an average diameter of 3–4 cm. Furthermore, the larynx (located higher in the pharynx) is fixed tightly to the trachea and can act as a “plug” thanks to its larger diameter (6–8 cm); thus, shape-locked gripping can be achieved (i.e., if the trachea is grasped tightly in the right position, then it cannot slip out because of the increasing diameter). However, if the preceding cuts were inaccurate (which should be hypothesized when using AI), then large amounts of surrounding soft tissue may remain on the trachea, making its shape and size uncertain. In this case, the gripper’s clamping force should be high enough to squeeze the soft tissue and reach the desired shape-locked state.

The secondary tasks of the gripper are the grasping, stretching and general manipulation of the legs to assist with the leg-cuttings. The usual diameter of the legs at the ankle is close to the diameter of the trachea (5–6 cm); thus, a mechanical gripper may be able to grasp both objects in the same way. Furthermore, the diameter of the limbs of the pigs has a local minimum close to the ankle, and thus, shape-locked gripping is possible in this case as well.

In addition to the mechanical requirements derived from the original gripping tasks, the integration of the gripper into the robot cell (the MFC) imposes requirements toward the gripper design too. The gripper was applied onto an ABB IRB4600 robot (ABB Ltd., Zürich, Switzerland) and used its 24V GPIO ports for communication and as input power as well (a pneumatic gripper design was also a possibility, but a built-in electromechanical motor was more convenient). As the goal of our research was to design a completely automated robot cell, the opening and closing of the gripper need to be controlled by a computer through the robot controller. The robustness of the whole system was a key factor from the planning phase, and thus, any kind of wireless communication was prohibited and the use of the integrated GPIO ports of the robot was preferred.

In summary, the basic requirements for the gripper for the given tasks are the following:

- Reliable grasping of a cylindrical object with known position and orientation in space:
  - Diameter: 3–4 cm;
  - Might be a random mass of soft tissues, up to 6–8 cm across;
  - Slippery, wet surface;
  - Relatively high position and orientation error should be hypothesized;
- All parts of the gripper should withstand at least 400 N (tensile strength of the trachea);
- Made of food-grade materials;
- Easy-to-clean design; can be decontaminated with industrial methods;
- Can be applied onto an industrial manipulator robot as an end-effector (both mechanically and electronically);
- Smart functionalities:
  - Closing-force sensing and/or control;
  - Slip-detection;
  - Feedback to the robot cell regarding the grasping.

### 2.2. Mechanical Design

Based on the described gripping tasks and the review of the state-of-the-art gripping solutions for abattoirs (e.g., DMRI’s gripper, shown in Figure 2), it was concluded that a 2-finger gripper would be the ideal choice with rotating fingers facing each other. This concept with the right-finger design leads to robust gripping:

- The fingers can reach around, lock on and pull in the target object within a sufficient diameter-range;
- The encircling motion allows compensation for uncertainty in the gripper-positioning (displacement of the leg/trachea in a radial direction and angular error);
- The design is modular, the shape of the fingers can be optimized for future tasks as well, as they are easily replaced;
- Gripping force can be measured and controlled relatively easily compared to soft grippers.

**Figure 5:**
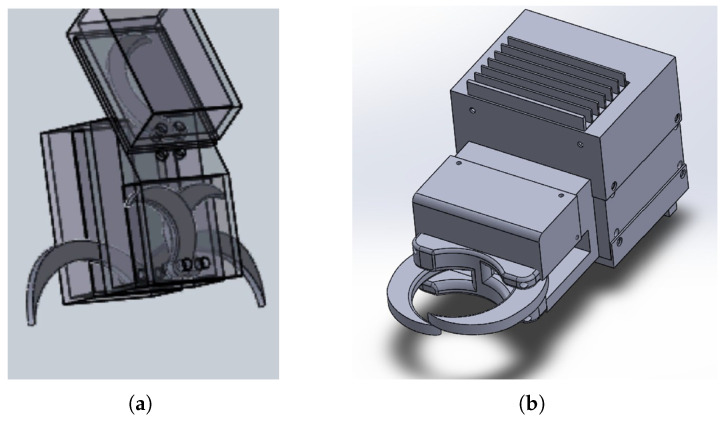

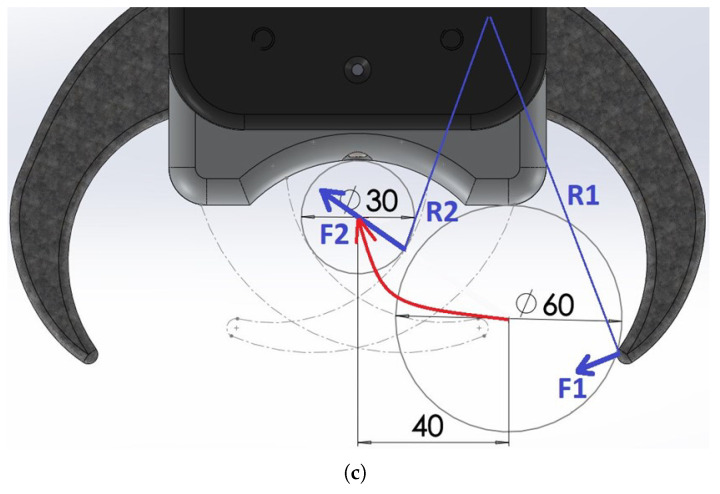
CAD models constructed during the design process of the gripper prototype. (**a**) The 1st conceptual design with 2 × 2 fingers and a cutting blade. (**b**) The final CAD model of the 1st prototype. (**c**) The finger design allows the grasping of objects up to 60 mm in diameter, with up to 40 mm radial displacement. The red arrow shows the constrained motion of the target object due to the synchronized encircling motion of the fingers. Furthermore, due to the constant closing torque and the decreasing lever arm (R2 < R1), the clamping force increases (F2 > F1) when the soft tissue is squeezed between the fingers, ensuring that a shape-locking grasp is achieved.

The first virtual design (CAD model) focused only on the described finger design, observing the physical reach requirements and position error tolerances. As shown in Figure 5, the mechanism is able to grasp objects up to a diameter of 60 mm with radial displacement up to 40 mm. Additionally, the synchronized encircling motion of the fingers forces the target object to the center line of the gripper, thus providing exact spatial coordinates of the trachea regardless of the estimation error of the trachea-locating image-processing neural network (which helps with the cutting of the trachea, which is the next step after it is grasped).

In addition to verifying finger design, the other objective of the 1st gripper prototype was the real-life examination of the fail-safe power transmission system of the device. The stepper motor (2 phase, 0.56 Nm, 12 W at this phase) was placed at the back of the gripper, its shaft connected to a worm gear (through an elastic shaft coupler), while the gripping fingers were fixed on the shafts of the two connecting worm wheels. The worm gear system has two main advantages in this application: it can provide a high transmission ratio by itself, and it is a self-locking mechanism, meaning that the gears cannot be driven from the side of the worm wheel. In this case, this means that only the motor can make the fingers move, and the gripper cannot be opened (or closed) by external forces acting on the fingers. This is beneficial when the intestines have to be pulled out of the belly by the gripper (tearing the connective tissues) since no additional energy input is needed after a firm grasp on the trachea is realized. Additionally, the self-locking mechanism provides fail-safe operation as well, since it means that even at a sudden power loss, the gripper maintains (most of) the clamping force, ensuring that the grasped target object will not fall out of the gripper. Furthermore, thanks to the high transmission ratio provided by the worm gear (1:30), the rotation of the fingers can be strong enough without additional gearboxes.

### 2.3. Final Gripper Design

The second gripper prototype was developed based on the experience of the described demo-cuts with the first prototype. Simulations using RoboDK (v5.1, RoboDK Inc., Montreal, QC, Canada) and RobotStudio, and rapid prototyping of the fingers, the housing and the whole gripper were used to test for dexterity, robot singularities and general workspace optimization and optimize the shape and dimensions. The important changes and additional features include the following (see Figure 5):

- Elongated shape and an additional spacer for robot workspace optimization;
- Completely sealed design;
- Optimized finger-shape;
- Stronger motor;
- All electronic components are soldered on dedicated, manufactured PCBs;
- Different novel integrated gripping force sensing solutions implemented;
- Smart features implemented: slip detection, force control, 2-way communication with the robot cell.

In summary, with respect to the mechanical aspects, the second version of the gripper became sleeker, adapting better to the task and the environment, and its overall dimensions became 70 × 100 × 350 mm. The gripper is mechanically separated into two easily detachable parts—the rear part contains the stepper motor and the power electronics, and the frontal one contains the sensors, the controller, the camera, etc. In addition to the worm gear, an additional internal gear was added with a 20:61 transmission ratio, resulting in a total of 2:183 torque-increasing transmission ratio between the motor shaft and the fingers. A significantly stronger motor (1.9 Nm instead of the 0.56 Nm) was chosen to provide a more reliable grasp when a large amount of tissue remains around the trachea, but thanks to the gripping force feedback, the chance of tissue damage due to too strong grasp remained low. The connections between the parts of the housing are sealed, and the moving parts are designed with simmering sealing as well; thus, the gripper is cleanable with standard industrial methods.

### 2.4. Smart Features

The final gripper prototype is a smart gripper thanks to the implemented sensors and computed modules and algorithms, which means that the gripper is capable of low-level, sensor-based decision-making as an integrated system. The gripper has two integrated processing units—an ESP32 microcontroller (ESP-WROVER-E from Espressif Systems) and an original Raspberry Pi Zero W. The microcontroller is responsible for the lower-level tasks (with supplementary chips), including motor speed control and signal processing of the different sensors. The Raspberry Pi manages high-level functionalities, including communication with the robot controller, external computers and the microcontroller; image processing of the endoscopic camera; and logging of internal states and sensory data.

#### 2.4.1. Force-Controlled Gripping

**Figure 6:**
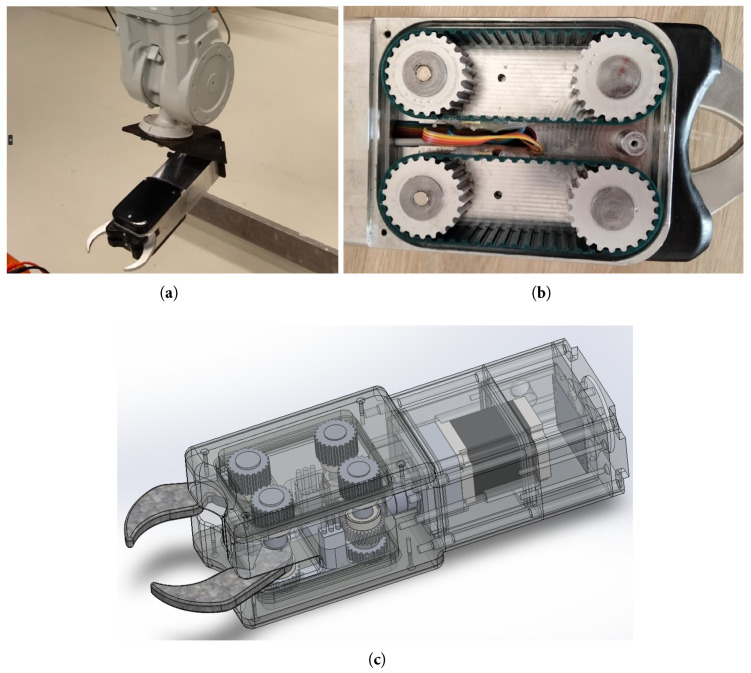
The final gripper model attached to the ABB IRB 4600 robot (**a**), its CAD model with improved finger design (**c**), and the timing belts in the power transmission system (**b**). The wires in the middle of the gripper transmit the sensory data of the strain gauges to the microcontroller.

The most advanced smart feature of the gripper is the clamping force control system that relies on novel clamping force estimation methods. The mechanical power transmission system of the gripper includes 2–2 ribbed belts, connecting the shafts of the fingers to the gearbox (Figure 6). Therefore, the force transmitted by the belts is proportional to the clamping force exerted by the fingers. As shown in Figure 6, the power transmission lines of the two fingers are mechanically separated (each finger is driven by independent belts); thus, the forces exerted by the fingers can be measured separately at this point of the drive chain.

The clamping force estimation method proposed in this paper is based on deformation measurements. The ribbed belts are steel cord-reinforced polyurethane belts (16 mm wide on one side, 10 mm wide on the opposite side for each finger, 260 mm long, T5 profile). It can be hypothesized that the steel cords take the vast majority of the transmitted force in the belts; thus, the stress–strain curves of such belts are close to the mechanical behavior of steel wires, i.e., the curves are approximately linear. Tensile testing of the belts with a tensile testing machine (H&P WPM Retrofit 400 KN (by Quantify, Tienen, Belgium), 5 independent measurements) proved the theory of linearity with R > 0.99 for all the tested belts (in the elastic region). Hypothesizing that the polyurethane “matrix material” and the cords form a legitimate composite together, the deformation of the belts should be directly proportional to the tensile stress on the belts, which is described by Hooke’s law:

(1) σ=E·ϵ,

where *σ* denotes tensile stress, *E* denotes the elastic modulus of the steel wires and *ϵ* denotes mechanical strain. Hooke’s law can be transformed to give the pulling force (*F*) as the result depending on the elongation of the belts (Δ*l*) using the active cross-section (i.e., the sum of the cross-sections of the cords, ∑*A_cords_*) and the initial active length of the belts (the distance of the shafts, in this case, *l*):

(2) $$F=\sum A_{cords}\cdot E\cdot 1/l\cdot \Delta l.$$

When the gripper is closing in on a target object, the active side of the belt drive (where tensile stress is present) is the one toward the midline of the gripper (i.e., in Figure 6, the upper gears are rotating clockwise during closing, the input side is the left side, and the fingers are on the right side). In summary, the elongation of the active section of the belt is directly proportional to the force exerted by the fingers on the target object, that is, the clamping force. Additionally, since the self-locking worm gear mechanism is located “behind” the belts from the fingers’ perspective, this design is capable of measuring the external forces acting on the gripper fingers as well, even when the gripper is stopped (according to Newton’s 3rd law). The limitation of this method is that the nonlinear effect of dry friction at the sealing of the fingers’ shafts produces elongation on the belt as well; however, it is mainly an offset error that can be easily handled by the controller software.

Strain gauges are widely used for elongation measurement of metals, but their operating range typically allows a maximum of 5% deformation; therefore, they usually cannot be used on polymers. However, in the case of steel cord-reinforced polyurethane belts, the elongation is maximized by the steel cords, and thus, strain gauges applied onto the polyurethane matrix material produce a sufficient method for belt elongation measurement. In most applications, 4 similar strain gauges are connected in a Wheatstone bridge, resulting in significant signal amplification and thermal compensation. Accordingly, “full bridge strain gauges” were chosen that consist of 4 strain gauges in a Wheatstone bridge connection on the same sheet, but the 2–2 gauges are oriented perpendicular to each other. Thanks to this orientation, the full bridge strain gauge can be attached on the belt in a way that 2 gauges are parallel to the belt’s direction of movement and 2 gauges are perpendicular to it. This means that the parallel gauges measure the elongation of the belts, while the perpendicularly oriented gauges do not react to the deformation, they just perform thermal compensation.

Early experiments with strain gauges applied on the belts resulted in a relatively high noise-to-signal ratio, and it was concluded that the accuracy of this method is highly dependent on the quality of the fixation. For this reason, another clamping force estimation method was developed, which is based on the measurement of the angular positions of the shafts. This method utilizes the linear elastic mathematical model of the belts given in (2) as well; however, the elongation is calculated from the rotational difference of the 2 belt-gears of each finger. When the motor starts rotating the input gears in the belt drive (i.e., the left side gears in Figure 6), the output gears start rotating as well; however, due to the elastic behavior of the belts, the rotation of the output gears are reduced by the elongation of the belts:

(3) $$\begin{array}{c} \phi_{2}=\phi_{1}-\Delta l/r,\\ \Delta l=(\phi_{1}-\phi_{2})*r, \end{array}$$

where Δ*l* is the elongation, *r* is the radius of the gears (20 mm) and *ϕ*_1_ and *ϕ*_2_ are the angular positions of the input and output gears, respectively. (3) means that the elongation (thus, the clamping force as well, according to (2)) is directly proportional to the difference between the angular positions of the gears connected by a belt and independent of the angular velocity or the actual angular position. The angular positions of the shafts are measured by magnetic encoders (As5601) with magnets fixed on the shafts. The resolution of the 12-bit contactless sensor is about 0.0015 [rad], meaning 30 µm of belt elongation. In addition to elongation calculation, the magnetic encoders are utilized to determine the angular position of the gripping fingers as well, which can indicate faulty gripping, e.g., if a high clamping force would be measured before the “closed” finger position was reached.

#### 2.4.2. Slip Detection

An additional smart feature of the gripper is the integrated slip detection algorithm. Slip detection is a key feature of any automation or robotics development that includes gripping. This is particularly true in the food industry as the target objects are typically intended for human consumption and—due to potential contamination–cannot be sold once dropped. Furthermore, slippage is a common problem in food industry automation due to slippery and wet surfaces, soft target materials, uncertain shapes and varying sizes.

**Figure 7:**
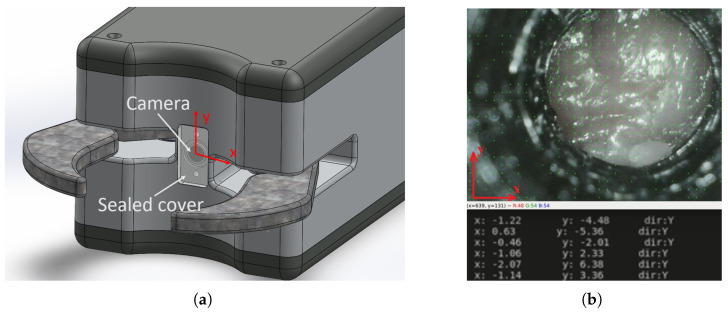
The smart slip detection system of the gripper. The camera is placed at the front of the gripper, and the synchronized encircling motion of the fingers ensures that the grasped object is pressed to the protective lens in front of the camera. The visualized optical flow field of the circular ROI shows the direction of the movement too. (**a**) Placement of the endoscopic camera at the front of the gripper behind the protective lens. (**b**) Typical camera frame with visualized optical flow and adjacent parameters computed.

To detect slippage, and furthermore, to potentially prevent the dropping of the grasped object, the gripper was designed with an integrated endoscopic camera (resolution: 640 × 480, 30 Frames per Second (FPS), 2 cm focus, 70° view angle, built-in LEDs). The camera is placed at the front of the gripper, as shown in Figure 7a. Thanks to the centralizing effect of the synchronized motion of the fingers, the grasped object is guaranteed to be pressed against the sealed polycarbonate cover plate in front of the camera. Although this design does not ensure a clear and sharp image, the cover plate stays clean enough for optical flow calculation thanks to the mandatory frequent washing of the whole gripper. The endoscopic camera is placed 2 cm behind the frontal surface of the cover plate, and thus, the surface of the grasped object is always in the focal plane of the camera. Six built-in LEDs around the camera lens provide sufficient illumination, even when the grasped object blocks all external light.

The video feed of the camera is processed internally by the Raspberry Pi Zero W. The slip detection algorithm was written in Python and built on top of the OpenCV library. The real-time image processing application relies on optical flow calculation to detect slippage of the grasped target objects. Optical flow algorithms are elementary tools for video processing, primarily for movement detection and movement-based object segmentation [29]. The fundamental assumption of optical flow is that the luminous intensities of the pixels on the observed objects do not change within an infinitesimal time during motion. This is not necessarily true, in general; however, thanks to the internal illumination from the built-in LEDs, it is a proper assumption in this case.

The slip detection algorithm uses the Farneback optical flow method, which has relatively high accuracy and sensitivity and low computing capacity requirements [30]. A typical frame from the slip detection camera during operation is shown in Figure 7b, and the calculated optical flow data are visualized on the image with green arrows in some grid points. It is visible that the optical flow of the black tube—in which the endoscopic camera is fixed—is zero in every grid point (only green dots can be seen); however, vectors with close to vertical orientation are visible on the piece of meat in the circular Region of Interest (ROI), indicating vertical movement, i.e., slippage. Below the frame, the simplified real-time numerical optical flow data are shown with the average of the X and Y components in the ROI and the direction of the detected movement when X or Y is clearly dominant.

Optical flow algorithms typically produce matrices of two-dimensional vectors for each pixel in each frame. However, for this application, where real-time operation on less powerful hardware is essential, the approach has been simplified. The video input resolution was lowered, and optical flow calculations were confined to a predefined ROI, measuring about 100 × 100 pixels at the given configuration. This adaptation with 30 FPS enables the optical-flow algorithm to operate in real-time on the integrated Raspberry Pi Zero W compute module. Although the target objects are soft and flexible, it was assumed that the piece of meat in the ROI is so small (less than 1 cm^2^) that it can be handled as a rigid body, meaning that the velocity (i.e., the optical flow) is roughly the same on the whole visible area. Given this assumption, the optical flow field is replaced with the calculated average optical flow vector in the ROI.

This simplified result of the optical flow calculation is processed internally and sent to the robot cell as well. Although the mean optical flow vector is recorded throughout the whole cutting process, the important output for the robot system is binary: whether slippage of the object is detected or not. The robot cell receives this feedback from the gripper through a GPIO port but can only react by sending a warning signal to the supervising operator. On the other hand, the gripper itself can react to slippage internally by increasing the clamping force, although tissue integrity has to be ensured as well.

The slip detection algorithm ran smoothly and the results were saved during several trial pig cuts, but the output has not been used in real-time yet. The main aspect of these workshops was the system integration of the Meat Factory Cell and fine-tuning of the AI-based software; thus, the log files of the gripper were analyzed retrospectively. During these tests, only a few slippages occurred, and these were all detected by the software.

## 3. Results

### 3.1. Mechanical Design

The prototype gripper was subjected to laboratory dry tests as well as on-site tests with real pig carcasses. First, the torque output (i.e., the clamping force) was computed by measuring the force acting between the two fingertips during closing with a calibrated load-cell. The highest measured force was 19 N (that means 1.7 Nm on the shafts of the fingers); however, the target objects should not get stuck between the fingertips. A 3 cm diameter object, when pressed to the base of the gripper (such as the 30 mm circle in Figure 5c) touches the active gripper finger at one-third of its length; thus, the maximum clamping force on such an object is around 60 N.

Furthermore, static load tests were performed on the gripper before the on-site tests with a 30 kg payload attached to the fingers. Non-sympathetic testing was even performed with much larger payloads, up to 900 N forces. In the beginning, the fingers’ axes of rotation were parallel to the gravitational force, and then, the gripper was rotated until the axes were perpendicular to it. The gripper was capable of lifting and holding the payload in any orientation without mechanical failure.

**Figure 8:**
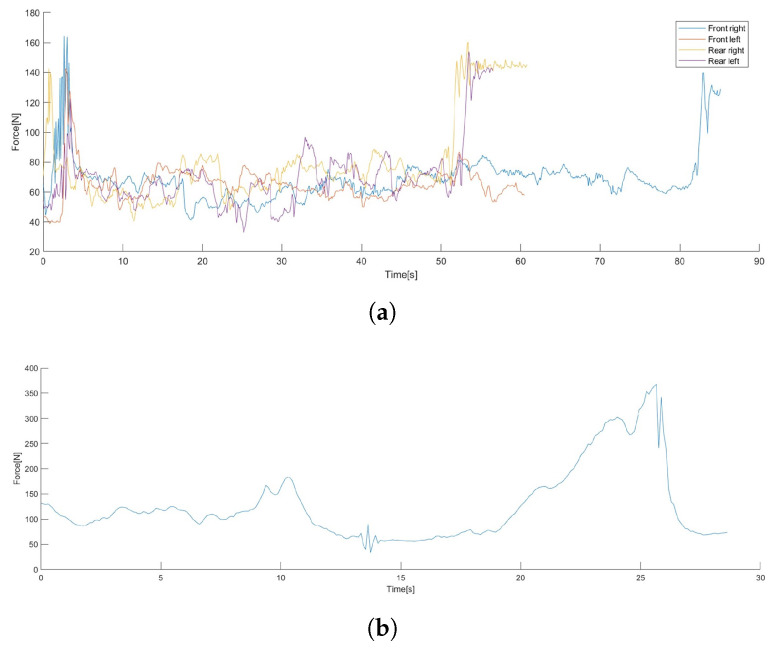
Resultant force data acquired during manual pig cuttings with the gripper prototype. (**a**) Plot of the forces measured during the cutting of the 4 limbs. (**b**) Plot of the forces acting during the evisceration process by pulling the trachea without any cuts made. At the peak force value (about 350 N at T = 26 s), the trachea cracked, resulting in some oscillation and then a steep fall; thus, this value became the maximum payload for further mechanical calculations.

The purpose of the on-site tests was to experiment with the gripper’s functionalities but also validate the novel automated evisceration process (i.e., that all the intestines can be pulled out with the gripper by the grasp on the trachea with minimal cutting). The gripper was mounted on a 6 DOF force torque sensor to acquire the maximal forces and torques during the processes (HEX-E/H QC by OnRobot A/S, Odense, Denmark, normal capacity: 200 N, 10 Nm (*x* and *y* axes), 6.5 Nm around the *z* axis). The first graph in Figure 8 shows the resultant force acting on the gripper during leg cuts. The graph starts when the fingers are already closed on the leg, and the short oscillations with 80–100 N amplitude at the beginning of the graphs indicate the initial pulling and jerking on the legs by the gripper, making sure that the grasp is tight and secure. The graphs start at around 40 N since the presented data are the raw resultant force; thus, the weight of the gripper itself (close to 4 kg) is still included. The final values of the graphs indicate the total weight of the cut limbs (plus the weight of the gripper) since they were lifted and moved to their basket using the gripper (except the front left leg, which was just released).

The second graph in Figure 8 shows the measurements when the diaphragm was already cut through and all the intestines were attempted to be removed only by pulling the trachea using the gripper. However, at a certain point—around T = 26 s on the graph—the trachea cracked and ripped off the pluck from the intestines. At that point, more than 350 N was measured as the resultant force, which thus can be called the tensile strength of the trachea (the breaking of the trachea was intentional in this case, and the proposed evisceration process was carried out successfully several times). This experiment showed that both the gripper and the trachea withstand the weight of all the inner organs and the legs (15–25 kg), and that the gripper is stronger than the organs, which means that it shall not break during these tasks under any circumstances.

### 3.2. Smart Features

**Figure 9:**
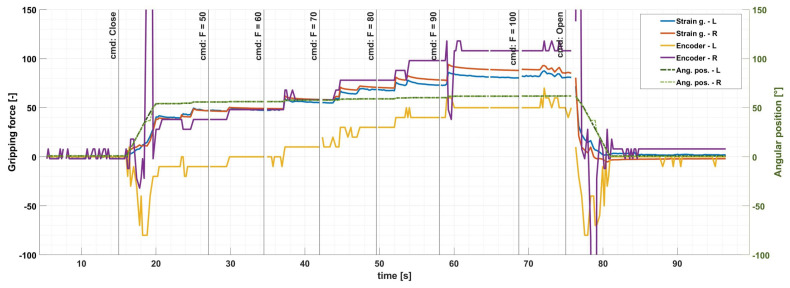
Force limitation mode test—exemplary data. The target force value is incremented by 10 N and denoted by the vertical black lines. The solid lines indicate the clamping force on the target object placed between the two fingers, measured by the 4 sensors (L and R denote sensors of the left and right side fingers, respectively), and the dotted lines denote the angular positions. The unit of the left-side Y axis strongly correlates to Newtons; however, due to uncompensated non-linearity at higher forces, the use of standard units might not be accurate.

**Figure 10:**
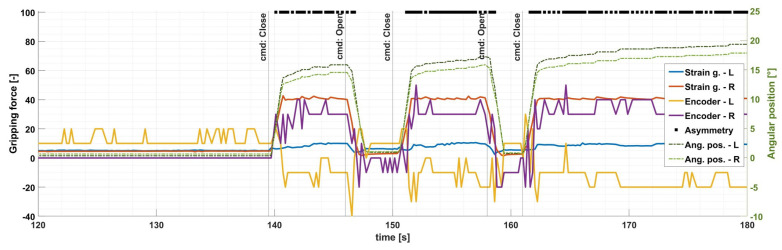
Graph of the clamping force measured on the fingers, and angular position measurements when the target object is off-center. The object (a pig’s leg) is initially positioned to the right; hence, the right-side force values increase while the left-side values merely become noisy (due to the mechanical connection between the two sides). The black lines at the top of the graph (labeled “Asymmetry”) denote the time-points when the device detected asymmetry and triggered a real-time warning to the system. The unit of the left-side Y axis strongly correlates to Newtons; however, due to uncompensated non-linearity at higher forces, the use of standard units might not be accurate.

The processing unit of the gripper handles both force estimation methods for the two fingers, meaning altogether four real-time, continuously submitted force values and two angular positions during operation. The purple and yellow lines in Figure 9 and Figure 10 show encoder-based force estimation while the red and blue lines show strain gauge-based data.

Figure 9 shows typical force estimation signals by the four methods and the angular positions of the fingers. The sensors were calibrated beforehand to show clamping force in Newtons (N); however, due to uncompensated non-linearity in the system (mainly originating from dry friction from sealings), the higher force values might have some deviation from the standard units; hence, it is not used on the vertical axes.

It can be seen in Figure 9 that the resolution of the encoder method is about 10 N (0.1 Nm on the shafts of the fingers). The resolution of the strain gauge method is much better (below 1 N); however, its nonlinear behaviors (e.g., relaxation) limit this method’s precision. Since the force estimations of the two different methods are relatively close to each other, their different characteristics can be utilized to reach a better, combined precision with the right mathematical signal processing methods.

The graph in Figure 9 shows the testing of the “clamping force limitation mode” of the gripper, which means that the gripper is closing until a given target clamping force is reached. and then stops. In this mode, when the clamping force decreases, the gripper closes further; however, it does not open when the measured force goes higher than the target value. The gripper can also operate in real-force-controlled mode with a P-type linear controller; however, the force limitation mode is more effective in the gripper’s use case.

Figure 10 shows the sensory data acquired during a real pig cutting using the entire robotized MFC setup. The data were recorded during a trial cut when a 3–4 cm gripping point calculation error was simulated (i.e., the robot operator guided the gripper to the leg of the pig with an intentional radial error). In this case, the right gripper finger contacted the leg earlier and reached the target force fast, but that was not high enough to pull the leg to the middle of the gripper (three attempts can be seen on the graph). The asymmetry can be detected on the graph between the angular position values (green dashed lines) and the force values (red and purple for the right side, blue and yellow for the left) as well. The asymmetry-detecting software monitors the latter. The black marks on the top of the graph indicate the time points when the gripper sent out an error signal to the robot cell about the displacement error, including the direction. During the automated operation, this signal can be fed back to the robot controller to adjust the gripper’s position in runtime.

## 4. Discussion

In summary, the gripper was used for more than 10 pig evisceration processes and was validated for leg gripping and manipulation as well in the Meat Factory Cell. Besides its satisfying mechanical properties, the presented gripper provides smart features that have the potential to complete different gripping and manipulation tasks during pig cutting in a safe, controlled and reliable way. The gripper can be fitted to and powered by industrial robots, realizes two-way communication with the robot controller, and offers an optional wireless connection for development and debugging.

The integrated clamping force control methods estimate the gripping force based on the measurement of deformation in the internal power transmission system. The strain gauge-based method offers high resolution, yet introduces nonlinear effects, while the magnetic encoder-based method has lower resolution but better reliability. Despite these limitations, both force sensing methods are sufficient for closed-loop clamping force control of the gripper within a linear proportional controller. Further mathematical signal processing and filtering, or the adoption of more advanced nonlinear controller algorithms, have the potential to improve the performance of the system without changing the hardware; however, the gripper is capable of performing its intended tasks in its current state too. Furthermore, force estimation methods also provide useful feedback about gripper positioning and the state of the manipulation task to the robot cell and the human supervisor.

Another smart feature of the gripper is the real-time slip detection system. This consists of an endoscopic camera and image processing software that recognize slippage of the target object through optical flow calculation. The software can differentiate between the general movement of the target and real slippage, and using the clamping force controller, the gripper can act in closed-loop control and prevent slippage by tightening its grip.

**Figure 11:**
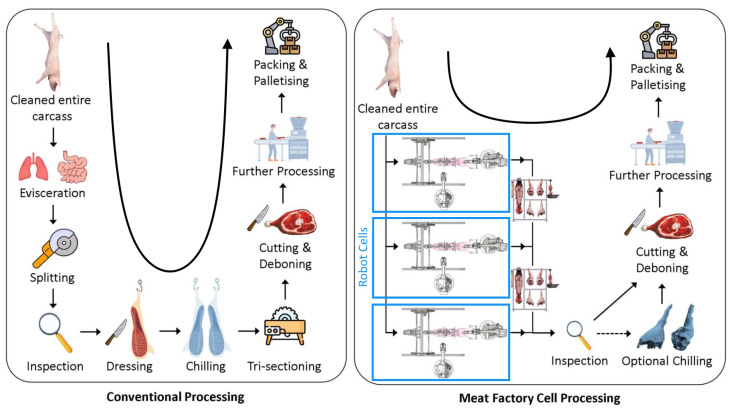
New concept of robotic red meat processing—the Meat Factory Cell (MFC) from the RoBUTCHER consortium. Based on Mason et al. [32].

Reconsidering the complete pig slaughter process under the scope of sustainability and ESG aspects is a tough challenge, partially addressed by the RoBUTCHER consortium [31]. Recent advances include the re-designed workflow and identification of the sensor system and the robotic and AI equipment necessary (Figure 11), explicitly stating the need for smart tools, such as sensor-enhanced grippers [25].

## 5. Conclusions

This paper presented a smart robotic gripper for meat industry automation purposes with an integrated clamping force control and slip detection system. The main intention was to build an intelligent meat industry gripper that is strong, cleanable and safe by design. Based on the experience gained during the early trials, the mechanical parts were strengthened and the motor was replaced; thus, the final design was able to safely and reliably grasp and manipulate the legs and trachea of the pigs. The device was initially designed for the RoBUTCHER project; however, the integrated novel solutions, design principles, or the gripper as a whole can be employed in other similar applications as well since, at present, no such intelligent general-purpose meat-industry gripper is available on the market.

The main smart features of the gripper are the clamping force control system and the slip detection algorithm. Both features are steps toward autonomous, yet still safe and reliable, handling of meat products, and furthermore, these provide important feedback to the meat factory cell about the gripping and manipulation tasks as well. The gripper was successfully integrated into the robot cell and was used several times for the evisceration process (trachea gripping), leg gripping and manipulation tasks. The trials showed that the clamping force control based on strain gauges adhered on ribbed belts, and the internal slip detection based on an integrated endoscopic camera are promising novelties toward more reliable meat industry gripping solutions. The gripper is made of food-grade materials and cleanable with industrial decontamination methods; thus, from the technical aspects, it has the potential to be used within automated meat processing plants on a larger scale.

## Data Availability

Data will be made available on request.

## Abbreviations

The following abbreviations are used in this manuscript:

AI	Artificial Intelligence
CAD	Computer-Aided Design
CHU	Carcass Handling Unit
DMRI	Danish Technological Institute
DOF	Degrees of Freedom
EOAT	End of Arm Tooling
EU	European Union
FPS	Frames per Second
GPIO	General Purpose Input/Output
MFC	Meat Factory Cell
NMBU	Norwegian University of Life Sciences
PCB	Printed Circuit Board
ROI	Region of Interest
RGB-D camera	Red-Green-Blue-Depth camera
